# Open reduction and internal fixation with cables for the variant A_GT_ Periprosthetic fracture: a case report and literature review

**DOI:** 10.1186/s42836-020-00029-5

**Published:** 2020-04-13

**Authors:** Meng-Qiang Fan, Xiao-Lei Chen, Yong Huang, Jie-Feng Huang

**Affiliations:** 1 Department of Orthopaedics, The First Affiliated Hospital of Zhejiang Chinese Medical University, 54 Youdian Road, Shangcheng District, Hangzhou, 310006 China; 2 The First Clinical College, Zhejiang Chinese Medical University, Zhejiang, 310006 Hangzhou China; 3 Basic Medical College, Shanghai University of Traditional Chinese Medicine, Shanghai, 201203 China; 4 Department of Orthopaedics, Hospital of Chengdu University of Traditional Chinese Medicine, 39 Shi’erqiao Road, Jinniu District, Chengdu, 610072 China

**Keywords:** Hip, Periprosthetic femoral fractures, A_GT_ periprosthetic fracture, Open reduction and internal fixation

## Abstract

**Background:**

Periprosthetic femoral fracture is identified as the third most frequent reason for revision total hip arthroplasty (THA). Treatment of periprosthetic fractures of the femur after THA remains a surgical challenge. In this report, we presented 2 patients with periprosthetic proximal femur fracture variant (a fracture of the greater trochanter with lateral cortical extension) and femoral stem destabilization.

**Cases presentation:**

Two patients presented with chief complaints of pain in hip, restricted hip movements and gait changes. On the basis of clinicoradiological findings, the patients were diagnosed as pseudo A_GT_ periprosthetic fracture, since the stem was loosened. They underwent open reduction and internal fixation (ORIF) with cables. After 2 years of follow-up, the 2 patients had favorable clinical outcomes after operation. Both lower limbs of the 2 patients were of equal length. The Harris score of the two hips was 96 and 94, respectively.

**Conclusion:**

CT scan worked better than X-ray examination in the diagnosis of prosthetic looseness with this type of fracture. Compared to longer-stem revision, ORIF with cables could also achieve good result with these fractures.

## Background

Periprosthetic femoral fracture (PPFF) is increasingly becoming a common complication of total hip arthroplasty (THA) and identified to be the third most frequent reason for revision THA [1]. With primary THA, the rate of intra-operative PPFF was reportedly 1.7% and a 20-year follow-up showed that the long-term rate was 3.5% [2].

Periprosthetic fractures are difficult to manage and may lead to poor outcomes. Post-THA treatment of PPFF remains a surgical challenge [3–5].

Presented in this report, were 2 patients suffering from periprosthetic proximal femur fracture variant (a fracture of the greater trochanter with lateral cortical extension), and femoral stem destabilization. The 2 patients had favorable clinical outcomes after open reduction and internal fixation (ORIF) with cables. Consents were obtained from the patients after they had been informed the fact their pictures might be submitted for publication.

## Case series

### Case 1

A 69-year-old man tripped and fell over. Subsequently, persistent pain developed in the right hip. Bilateral radiography of hips 2 h after the fall revealed femoral neck fracture of the right hip.

**Fig. 1:**
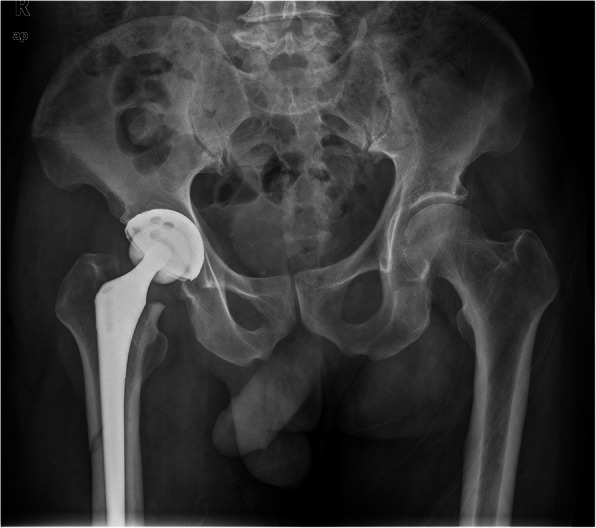
Anteroposterior radiograph of the right hip in postoperative day 1 showed the distal extension of the fracture line down the lateral cortex

**Fig. 2:**
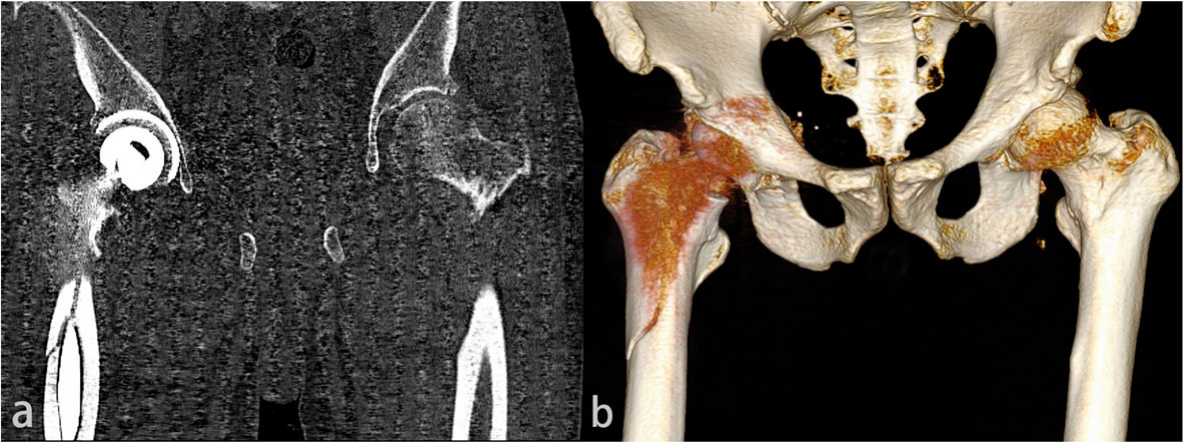
**a-b** Computed tomography scan and three-dimensional reconstruction of the right hip in postoperative day 1 showed the distal extension of the fracture line down the lateral cortex; this leads to destabilization of the stem because the lateral buttress is lost

The patient was taken to the operating room 1 day after injury for THA of the right hip. Anteroposterior radiography (Fig. 1) and computed tomography (CT) (Fig. 2) 1 day after operation showed that periprosthetic fracture of the proximal femur involved the greater trochanter, with lateral cortical extension.

**Fig. 3:**
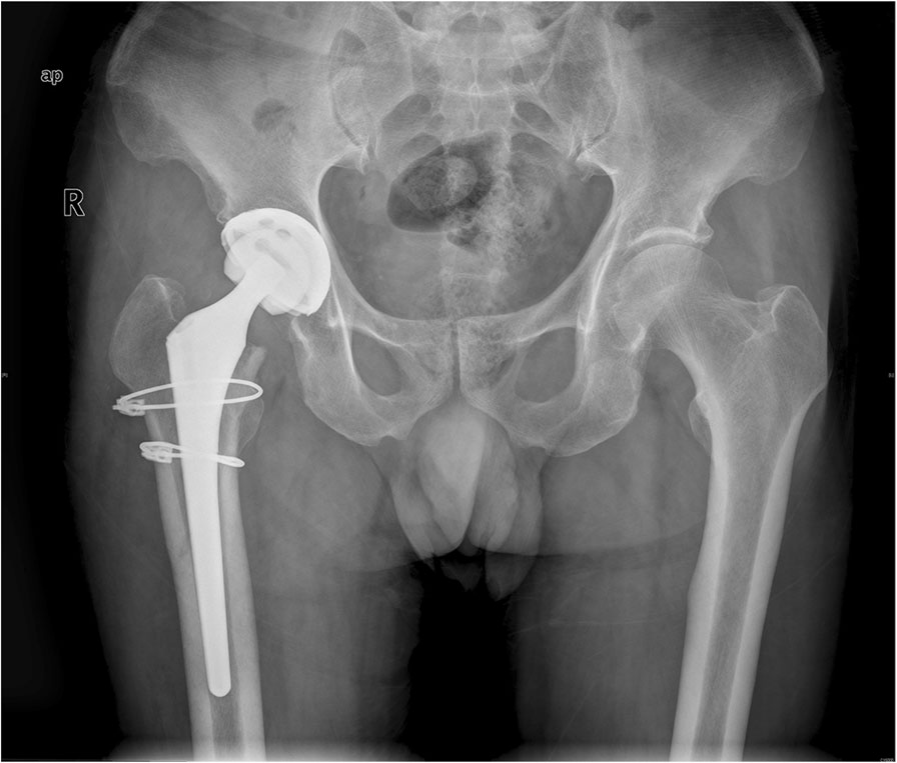
Anteroposterior radiograph 2 years after ORIF showed reduction and fixation of the fracture, the fracture healed well, and the stem is stabilized

ORIF was performed 2 days after the diagnosis, and the 2 cables were annularly fixed above and below the small trochanter separately. Anteroposterior radiography (Fig. 3) 2 years after surgery showed the fracture healed well, and the stem was stable. Both lower limbs were of equal length, and the Harris score of the right hip was 96.

### Case 2

**Fig. 4:**
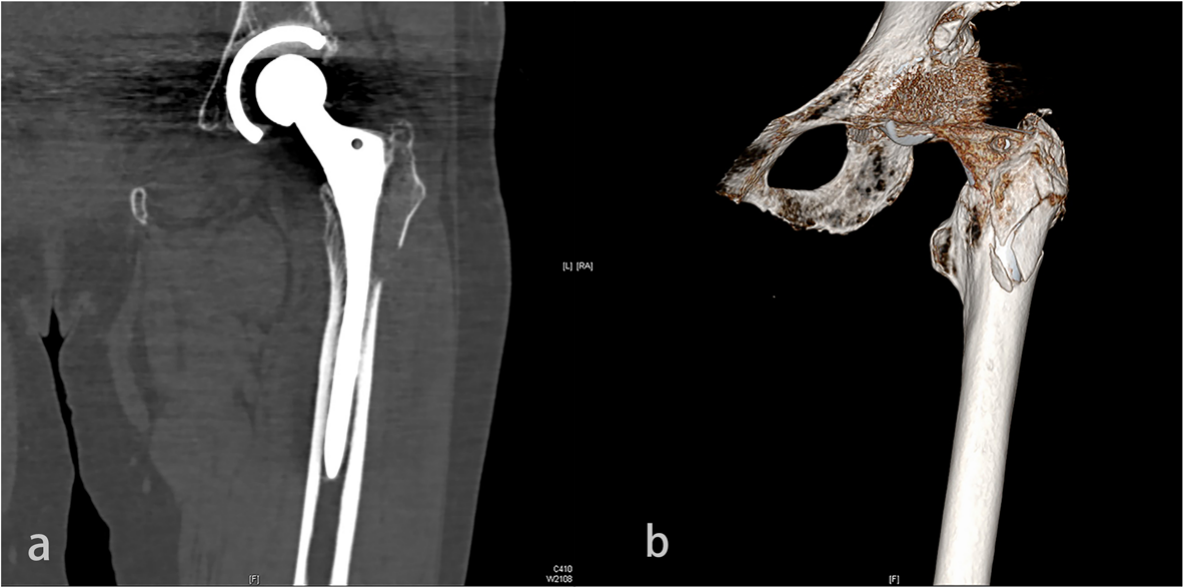
**a-b** Computed tomography scan and three-dimensional reconstruction of the left hip in postoperative day 1 showed the distal extension of the fracture line down the lateral cortex; this leads to destabilization of the stem because the lateral buttress is lost

An 82-year-old woman suffered from severe pain in her left hip after she fell over while walking. Bilateral radiography of hips 1 h after the fall exhibited femoral neck fracture of the left hip. The patient received THA of the left hip 2 days after the injury. CT scan (Fig. 4 a) and three-dimensional reconstruction (Fig. 4 b) 1 day after the operation showed that periprosthetic fracture of the proximal femur affected the greater trochanter and the lateral cortex of the proximal femur.

**Fig. 5:**
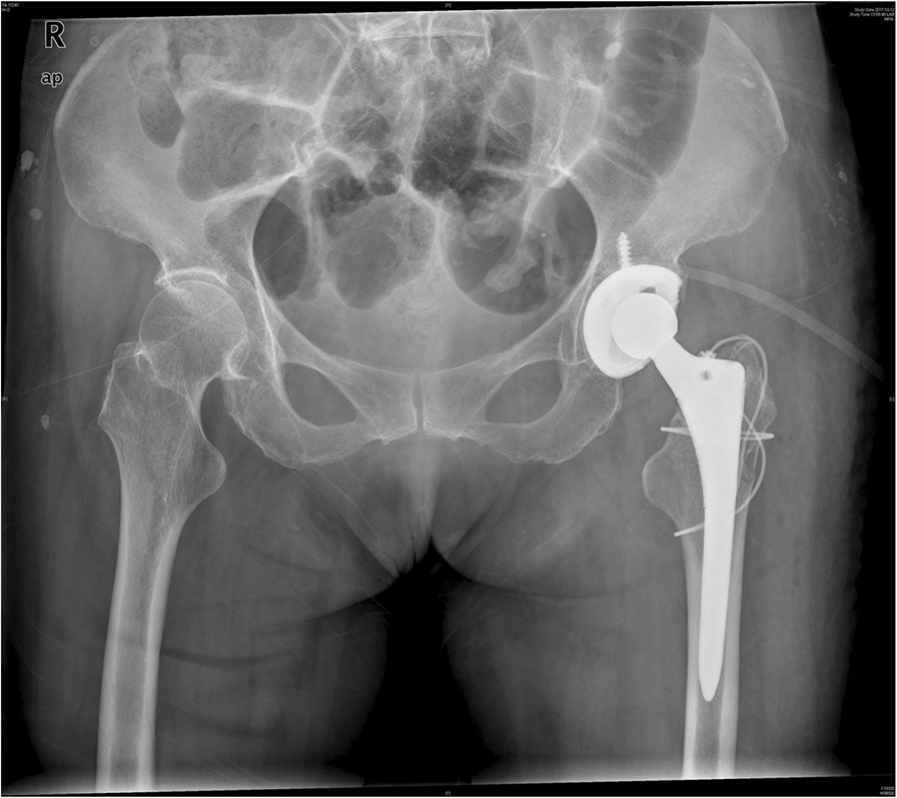
Anteroposterior radiograph 2 years after ORIF showed reduction and fixation of the fracture, the fracture healed well, and the stem was stabilized

The patient underwent ORIF 2 days after diagnosis. Two cables were used to fix the fracture, one of which was circumferentially placed above the small trochanter and the other was placed in a ‘8-shape’ from the large trochanter to the lower trochanter. Anteroposterior radiography (Fig. 5) 2 years after ORIF showed the fracture healed well, and the stem was stable. Both lower limbs were found to be of equal length, and the Harris score of the left hip was 94.

## Discussion

The Vancouver Classification System (VCS) [6] and the Unified Classification System (UCS) [7] for PPFF have been generally accepted. The VCS focuses on the location of the fracture relative to the stem, the stability of the implant, and the associated bone loss [6]. Type A fractures are in the trochanteric region, type B fractures involve the area of the stem, and type C fractures are distant from the tip of the stem. Duncan and Haddad [7] introduced the UCS to expand and update the VCS and apply treatment principles to all periprosthetic fractures. When applied to the femur, the UCS retains the previous VCS patterns and extends to include two new fracture patterns, type D and E. Type D refers to a fracture of the femur after hip and knee arthroplasty (Type C for each joint). Type E is a fracture involving both the acetabulum and femur after hip arthroplasty.

In both VCS and UCS, Type A fractures are subdivided into fractures of the greater trochanter (A_GT_) and those of the lesser trochanter (A_LT_). Van Houwelingen and Duncan [8], and Capello *et al*. [9] reported pseudo A_LT_ periprosthetic fractures that were actually Type B2 of VCS. This type of periprosthetic fracture of the lesser trochanter included a segment of the proximal medial femoral cortex. However, in this report, we presented 2 cases of periprosthetic fracture of the proximal femur involving the greater trochanter with lateral cortical extension, leading to destabilization of the stem. These periprosthetic fractures of the proximal femur involving the lesser/greater trochanter with medial/lateral cortical extension can be classified as variant Type A fractures that are actually Type B2.

**Fig. 6:**
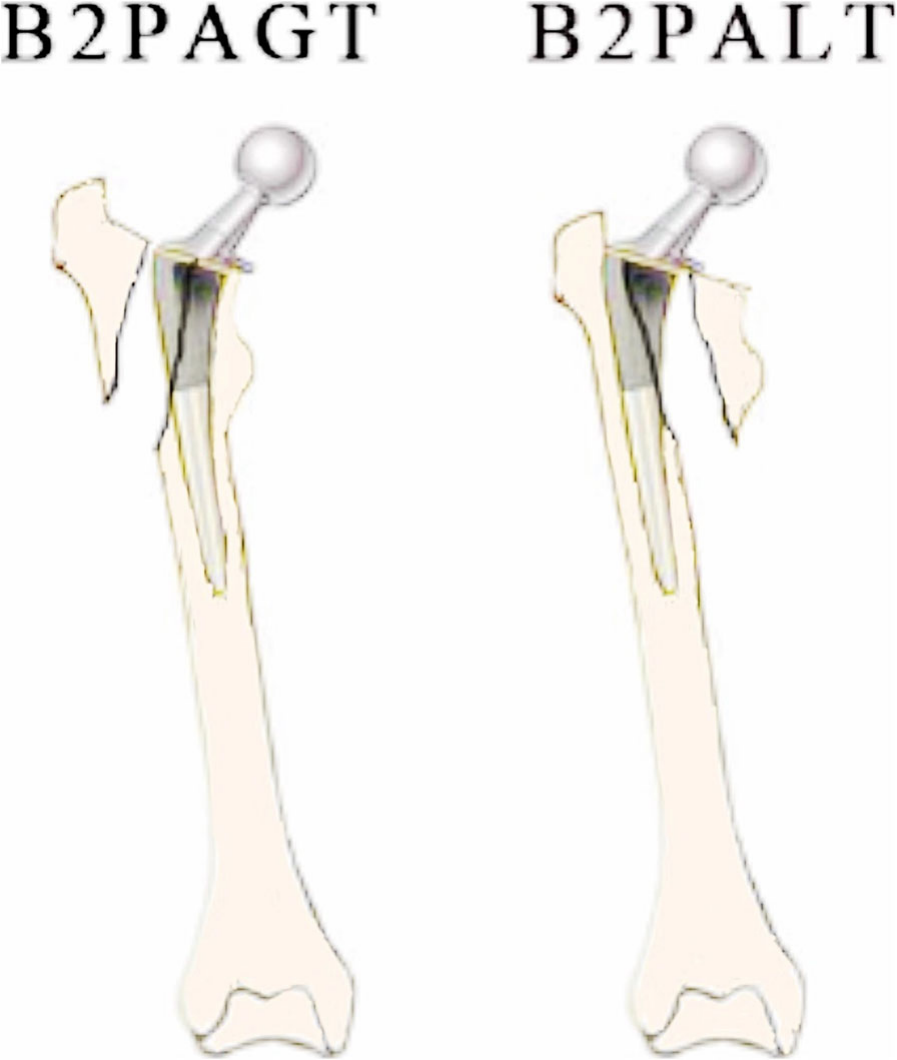
The Modified Unified Classification System [10]: B2PALT/B2PAGT was defined as fracture in trochanter region including a segment of the proximal medial/lateral femoral cortex

On the basis of a systematic literature review and an evaluation of 402 cases of PPFF, Huang *et al*. [10] introduced a more precise fracture classification based on the original UCS by (1) adding two new B2 subtypes: B2PALT (i.e., pseudo A_LT_) and B2PAGT (i.e., pseudo A_GT_) and (2) adding a new FS category to encompass stem fracture, alone or accompanied with PPFF. B2PALT/B2PAGT was defined as fracture in trochanter region that includes a segment of the proximal medial/lateral femoral cortex (Fig. 6). According to the modified UCS [10], the 2 cases in this report were categorized as B2PAGT.

It’s worth mentioning why this type is classified as “B2”. The key distinguishing feature between the type A_GT_ fracture and pseudo A_GT_ periprosthetic fracture of the greater trochanter lies in the distal extension of the fracture involving the lateral cortex of the proximal femur, which destabilizes the stem in a B2 fracture. CT scan can help clinicians to determine the stability of the stem and distinguish between fracture type A and type B. The region involved in this type of fracture (i.e., Baba classification Type 1A) can render the stem unstable [11].

The 2 variant Type A_GT_ fractures were both diagnosed 1 day after operation. This is usually seen within 6 weeks of the index procedure, typically following the insertion of a tapered, cementless stem within a demineralized femur. The mechanism may be due to an unrecognized intraoperative fracture that is subsequently displaced under load of muscular tension, or may occur immediately after or during rehabilitation.

The principles of treatment depend on the timing of the fracture and the size of the medial/lateral fracture fragment. If recognized intraoperatively as non-propagating cortical crack, then extraction of the broach or stem, followed by cerclage cable fixation and reinsertion of the stem is adequate in most cases, plus protected weight bearing for 6 weeks. Missed diagnosis or fractures that occur in the early postoperative period with associated fracture displacement and implant subsidence often require THA revision with a longer stem, along with ORIF of the fracture using cerclage cables and/or proximal femoral plating [8, 10].

However, we did not perform a revision with a longer stem, but just employed ORIF with 2 cables, with weight bearing starting from the day after surgery. The reason why we chose ORIF alone over revision THA with longer stem lies in that (1) The mechanism of injury in variant Type A_GT_ fractures is similar to Pseudo A_LT_ fracture [8]. (2) Not all the Type B2 fractures require THA revision. Capello *et al*. [9] reported 9 Pseudo A_LT_ fractures, whereas 3 of 9 cases were successfully managed non-surgically. In their study, the fracture had been noted early postoperatively, frequently with stem subsidence but needed no surgery and the stem restabilization, and subsequent surgery. (3) Our past experience with arthroplasty for unstable intertrochanteric osteoporotic fractures, along with ORIF of the fractures prompted us to use cerclage cables [12].

On the basis of about findings, we are led to conclude that early cerclage cable fixation alone, can successfully address this particular Vancouver Type A periprosthetic fracture variant despite reported destabilization of the femoral stem. Re-stabilization was based on the principle of stem subsidence. Although the principles of treatment suggest use of longer stem revision and the fracture fixation, ORIF has the advantages of minimal invasion and rapid recovery. In addition, we measured the stem position from the X-Ray films immediately after operation and in a two-year follow-up, and found that there was no significant stem subsidence or lower limb shortening.

## Conclusion

It is important to distinguish the variant type A_GT_ periprosthetic fracture from the type A_GT_, because type A_GT_ periprosthetic fracture is associated with destabilization of the stem and requires early re-intervention. CT scan works better than X-ray examination in finding prosthetic looseness in this type of fracture. These cases illustrated that ORIF with cable could, in some variant type A_GT_ periprosthetic fractures, achieve successful healing and stem stabilization.

## Data Availability

All data generated or analyzed during this study are included in this published article.

## Abbreviations

THA: Total hip arthroplasty
ORIF: Open reduction and internal fixation
PPFF: Periprosthetic femoral fractures
CT: Computed tomography
VCS: Vancouver Classification System
UCS: Unified Classification System (UCS)
