# Correction to: Open reduction and internal fixation with cables for the variant A_GT_ Periprosthetic fracture: a case report and literature review

**DOI:** 10.1186/s42836-020-00033-9

**Published:** 2020-05-05

**Authors:** Meng-Qiang Fan, Xiao-Lei Chen, Yong Huang, Jie-Feng Huang

**Affiliations:** 1 Department of Orthopaedics, The First Affiliated Hospital of Zhejiang Chinese Medical University, 54 Youdian Road, Shangcheng District, Hangzhou, 310006 China; 2 The First Clinical College, Zhejiang Chinese Medical University, Hangzhou, 310006 Zhejiang China; 3 Basic Medical College, Shanghai University of Traditional Chinese Medicine, Shanghai, 201203 China; 4 Department of Orthopaedics, Hospital of Chengdu University of Traditional Chinese Medicine, 39 Shi’erqiao Road, Jinniu District, Chengdu, 610072 China

**Correction to: Arthroplasty 2, 10 (2020)**

**https://doi.org/10.1186/s42836-020-00029-5**

In the original publication of this article [1], Fig. 5 needs to be revised. The updated Fig. 5 is shown below.

**Fig. 5:**
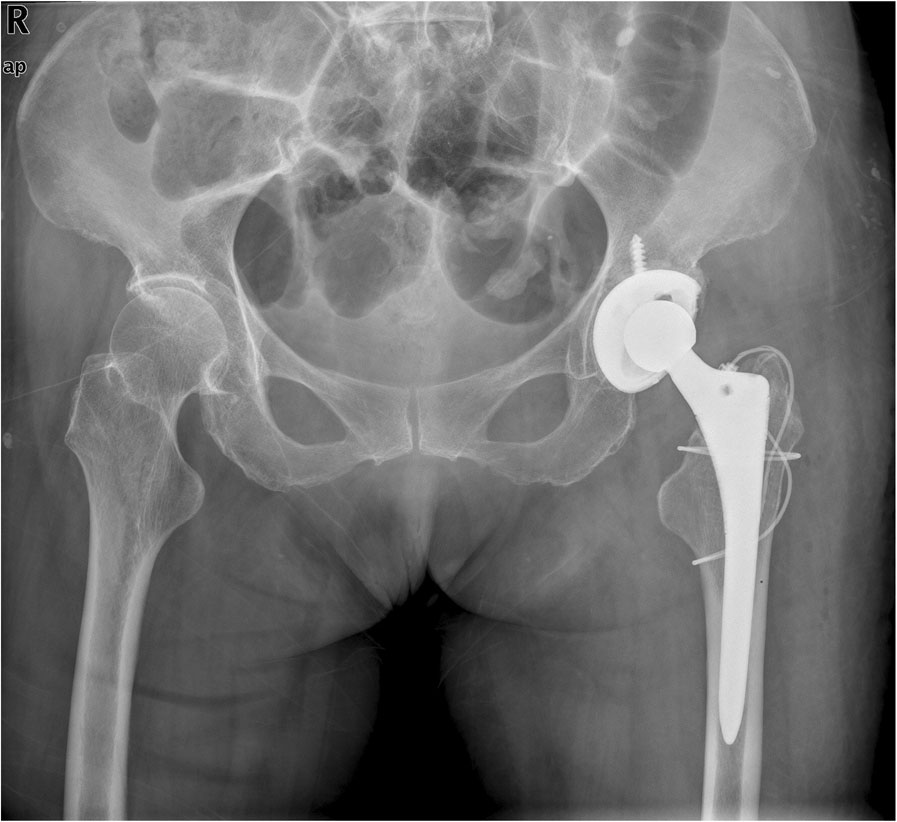
Anteroposterior radiograph 2 years after ORIF showed reduction and fixation of the fracture, the fracture healed well, and the stem was stabilized
